# A Finite Difference Algorithm Applied to the Averaged Equations of the Heat Conduction Issue in Biperiodic Composites—Robin Boundary Conditions

**DOI:** 10.3390/ma14216329

**Published:** 2021-10-23

**Authors:** Ewelina Kubacka, Piotr Ostrowski

**Affiliations:** Department of Structural Mechanics, Lodz University of Technology, 93-590 Lodz, Poland; piotr.ostrowski@p.lodz.pl

**Keywords:** composite, heat conduction, finite difference method, biperiodic structures

## Abstract

This note deals with the heat conduction issue in biperiodic composites made of two different materials. To consider such a nonuniform structure, the equations describing the behavior of the composite under thermal (Robin) boundary conditions were averaged by using tolerance modelling. In this note, the process of creating an algorithm that uses the finite difference method to deal with averaged model equations is shown. This algorithm can be used to solve these equations and find out the temperature field distribution of a biperiodic composite.

## 1. Introduction

The primary purpose of this research was to create a computational algorithm that can be used in the analysis of the heat conduction issue with regard to biperiodic composites. Periodic or functionally graded structures made of two or more materials can exhibit advantageous mechanical or strength properties (in view of the adopted criterion). For this reason, it is important to be able to consider and model these structures. There are many methods which can be used in the analysis of heterogeneous structures such as composites. Among them are asymptotic homogenization [1,2], homogenization with microlocal parameters [3,4], the finite element method [5,6], the boundary element method [7], the meshless methods [8], the higher order theory [9,10], and the methods using the Green–Lindsay model [11]. In the analyzed biperiodic structures, the so-called periodicity cells can be mentally separated. In turn, these cells are characterized by a certain dimension, called the microstructure size or microstructure parameter. Unfortunately, most known methods do not allow including this parameter in the considerations. Therefore, to obtain the equations describing the considered issue, tolerance modelling [12,13,14] was used. Then, a computational algorithm was created to analyze the heat conduction phenomenon in biperiodic composites, using the finite difference method on the derived equations. By using this algorithm, it is possible to obtain the temperature field distribution in the analyzed composite. On selected edges of this structure, the third kind boundary conditions were imposed (the Robin boundary conditions) [15].

**Figure 1:**
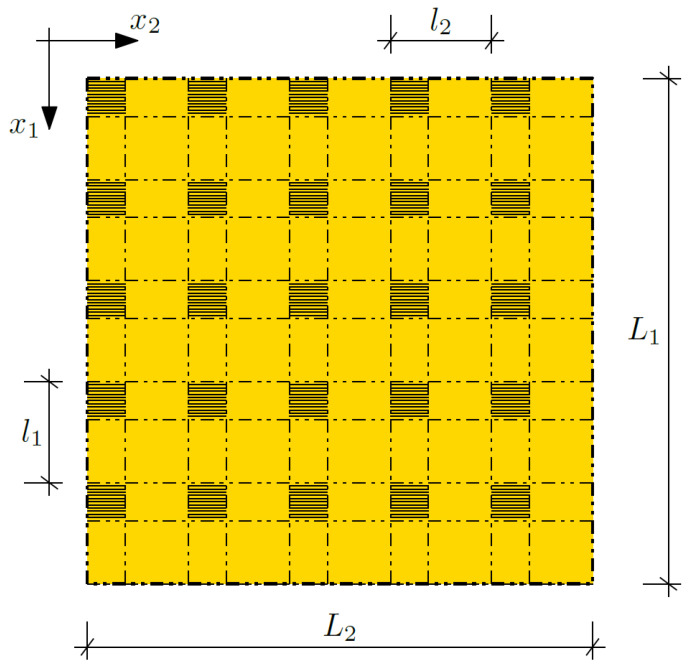
Biperiodic composite.

The theoretical structure we analyze herein is a composite made of two materials with different properties; confer Figure 1. These material properties are changing in a periodic way along both perpendicular directions (*x*_1_ and *x*_2_). The dimension along *x*_1_ is denoted by *L*_1_, and that in direction *x*_2_—analogously—by *L*_2_. The mentioned microstructure parameters along directions *x*_1_ and *x*_2_, are denoted by *l*_1_ and *l*_2_, respectively. The microstructure size is directly related to the number of composite layers (cells).

The volume share of each material in every cell is determined and constant. The product of the share of the first material in a cell along *x*_1_ and along *x*_2_ is a volume share of the first material in a cell and is denoted by ν_1_ = ν_1_(*x*_1_, *x*_2_). The volume share of the second material in a cell ν_2_ = ν_2_(*x*_1_, *x*_2_) can be calculated in an analogous way.

## 2. Averaged Equations

Equation (1) describing the heat conduction phenomenon with reference to biperiodic composite is an equation with noncontinuous coefficients:

(1) $$\partial_{i}\left(k_{ij}\partial_{j}\theta \right)-c\varrho \dot{\theta }=0,$$

where θ is an unknown temperature field; *c* and ρ specify the material properties such as a specific heat and a mass density, respectively; and *k_ij_* defines the components of the conductivity tensor. To average this equation, tolerance modelling was used [16,17,18,19,20,21,22].

**Figure 2:**
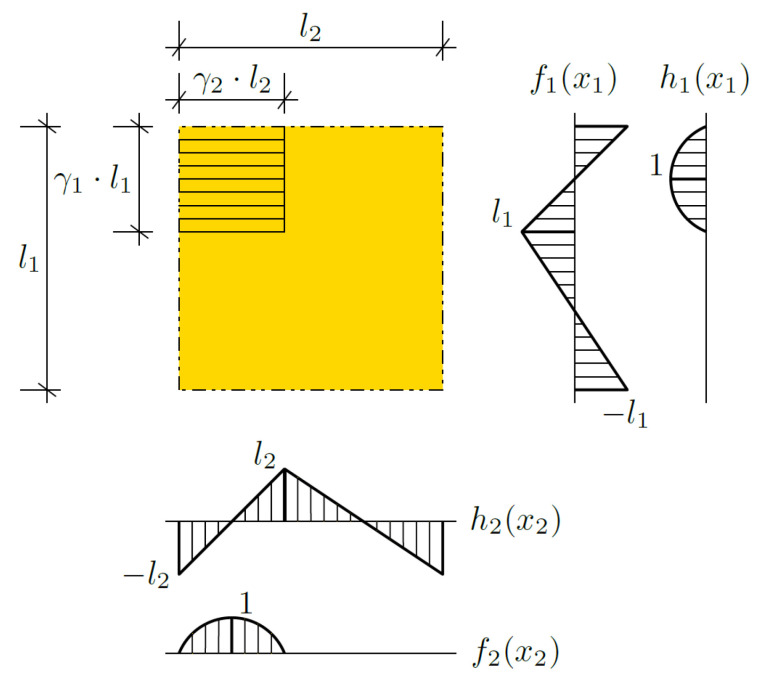
Fluctuation shape functions.

Tolerance modelling, also called the tolerance averaging technique, introduces in a process of modelling a new concepts, definitions, and assumptions. The new concepts primarily relate to functions that comply with certain conditions. Among them we can distinguish the tolerance-periodic function, the highly-oscillating function, and the slowly-varying function. Among the assumptions of this method, the most important, from the point of view of the present considerations, is the micro-macro decomposition assumption. In conformity with this assumption, the unknown temperature field can be taken according to the following formula:

(2) $$\Theta \left(x_{1},x_{2}\right)=\theta \left(x_{1},x_{2}\right)+g_{1}\left(x_{1},x_{2}\right)\cdot \psi_{1}\left(x_{1},x_{2}\right)+g_{2}\left(x_{1},x_{2}\right)\cdot \psi_{2}\left(x_{1},x_{2}\right),$$

where θ is an averaged part of the temperature field Θ, called the macrotemperature. The remainder of the equation is the sum of the products of the fluctuation shape functions *g*_1_ and *g*_2;_ and fluctuation amplitudes ψ_1_ and ψ_2_. The mentioned sum is called the fluctuating part of the temperature field Θ. The fluctuation amplitudes are the new unknowns, and the fluctuation shape functions are assumed a priori. These functions should be assumed individually, taking into account the specifics of the issues under consideration. In this note, it is important to adopt functions that guarantee the continuity of the total temperature, both between the layers (cells), and inside the cells on interfaces between materials. Therefore, some combination of the basic functions (saw-type and piecewise parabolic functions) shown in Figure 2 are assumed.

By γ_1_ and γ_2_, the shares of the first material in a cell in direction *x*_1_ and in direction *x*_2_, respectively, are denoted. The fluctuation shape functions *g*_1_(*x*_1_, *x*_2_) and *g*_2_(*x*_1_, *x*_2_) are assumed according to the following formulas:

(3) $$g_{1}\left(x_{1},x_{2}\right)=f_{1}\left(x_{1}\right)\cdot f_{2}\left(x_{2}\right),$$

(4) $$g_{2}\left(x_{1},x_{2}\right)=h_{1}\left(x_{1}\right)\cdot h_{2}\left(x_{2}\right),$$

*f*_1_, *f*_2_, *h*_1_, and *h*_2_ are depicted in Figure 2.

By considering the micro-macro decomposition assumption, and the other assumptions and definitions of the tolerance modelling discussed in [23,24,25], Equation (1) describing the heat conduction issue was averaged, leading to the tolerance model equations:

(5) $$\langle c\varrho \rangle \dot{\Theta }-\nabla \left(\langle K\rangle \nabla \theta +\langle K\partial g_{1}\rangle \psi_{1}+\langle Kg_{1}\rangle \bar{\nabla }\psi_{1}+\langle K\partial g_{2}\rangle \psi_{2}+\langle Kg_{2}\rangle \bar{\nabla }\psi_{2}\right)=0,$$

(6) $$\begin{array}{l} \langle c\varrho g_{1}\rangle \dot{\Theta }+\langle K\partial g_{1}\rangle \nabla \theta +\langle K\partial g_{1}\partial g_{1}\rangle \psi_{1}+\langle K\partial g_{1}g_{1}\rangle \bar{\nabla }\psi_{1}+\langle K\partial g_{1}\partial g_{2}\rangle \psi_{2}+\\ +\langle K\partial g_{1}g_{2}\rangle \bar{\nabla }\psi_{2}-\bar{\nabla }\left(\begin{array}{l} \langle Kg_{1}\rangle \nabla \theta +\langle K\partial g_{1}g_{1}\rangle \psi_{1}+\langle Kg_{1}g_{1}\rangle \bar{\nabla }\psi_{1}\\ +\langle K\partial g_{2}g_{1}\rangle \psi_{2}+\langle Kg_{1}g_{2}\rangle \bar{\nabla }\psi_{2} \end{array}\right)=0 \end{array},$$

(7) $$\begin{array}{l} \langle c\varrho g_{2}\rangle \dot{\Theta }+\langle K\partial g_{2}\rangle \nabla \theta +\langle K\partial g_{1}\partial g_{2}\rangle \psi_{1}+\langle K\partial g_{2}g_{1}\rangle \bar{\nabla }\psi_{1}+\langle K\partial g_{2}\partial g_{2}\rangle \psi_{2}+\\ +\langle K\partial g_{2}g_{2}\rangle \bar{\nabla }\psi_{2}-\bar{\nabla }\left(\begin{array}{l} \langle Kg_{2}\rangle \nabla \theta +\langle K\partial g_{1}g_{2}\rangle \psi_{1}+\langle Kg_{1}g_{2}\rangle \bar{\nabla }\psi_{1}\\ +\langle K\partial g_{2}g_{2}\rangle \psi_{2}+\langle Kg_{2}g_{2}\rangle \bar{\nabla }\psi_{2} \end{array}\right)=0 \end{array}.$$

where **K** stands for the thermal conductivity tensor whose components are *k_ij_*, ∇ is a gradient operator defined as (∂_1_, ∂_2_, ∂_3_), overlined ∇ is a gradient in *x*_3_ direction (0, 0, ∂_3_), and ∂ is a gradient operator defined as (∂_1_, ∂_2_, 0).

Equations (5)–(7) can be obtained by using the orthogonalization method [26] or by the extended stationary action principle [27,28].

The averaging operator, denoted in Equations (5)–(7) by triangular brackets, is defined according to the following equation:

(8) $$\langle \partial^{i}F\rangle \left(x\right)\equiv \frac{1}{\left|\lambda \right|}\int_{\lambda \left(x\right)}{\tilde{F}}^{i}\left(x,z\right)dz,$$

where an approximation of the function *F*(*x*) is denoted by symbol tilde ~; points ***x*** = (*x*_1_, *x*_2_) and ***z*** = (*z*_1_, *z*_2_) are, respectively, coordinates in the global and local systems; λ = (−*l*_1_/2,*l*_1_/2) × (−*l*_2_/2,*l*_2_/2) stands for the unit cell; and λ(***x***) = λ + ***x***.

## 3. Algorithm of Finite Difference Method

First, the equations of the tolerance model were reformulated and written in index notation. The indices take the following values: the superscripts *a*, *b* = 1, 2, the subscripts *a*, *b* = 1, 2, 3; and the subscripts α, β = 3:

(9) $$\langle c\varrho \rangle \left(\dot{\theta }+\langle g_{1}\rangle {\dot{\psi }}_{1}+\langle g_{2}\rangle {\dot{\psi }}_{2}\right)-\partial_{b}\left(\begin{array}{l} \langle k_{ab}\rangle \partial_{a}\theta +\langle k_{ab}\partial^{a}g_{1}\rangle \psi_{1}+\langle k_{\alpha b}g_{1}\rangle \partial_{\alpha }\psi_{1}\\ +\langle k_{ab}\partial^{a}g_{2}\rangle \psi_{2}+\langle k_{\alpha b}g_{2}\rangle \partial_{\alpha }\psi_{2} \end{array}\right)=0,$$

(10) $$\begin{array}{l} \langle c\varrho g_{1}\rangle \left(\dot{\theta }+\langle g_{1}\rangle {\dot{\psi }}_{1}+\langle g_{2}\rangle {\dot{\psi }}_{2}\right)+\langle k_{ab}\partial^{a}g_{1}\rangle \partial_{b}\theta +\langle k_{ab}\partial^{a}g_{1}\partial^{b}g_{1}\rangle \psi_{1}+\langle k_{a\alpha }\partial^{a}g_{1}g_{1}\rangle \partial_{\alpha }\psi_{1}+\\ +\langle k_{ab}\partial^{a}g_{1}\partial^{b}g_{2}\rangle \psi_{2}+\langle k_{a\alpha }\partial^{a}g_{1}g_{2}\rangle \partial_{\alpha }\psi_{2}-\partial_{\alpha }\left(\langle k_{a\alpha }g_{1}\rangle \partial_{a}\theta \right)+\\ -\partial_{\alpha }\left(\langle k_{a\alpha }\partial^{a}g_{1}g_{1}\rangle \psi_{1}+\langle k_{\alpha \beta }g_{1}g_{1}\rangle \partial_{\beta }\psi_{1}+\langle k_{a\alpha }\partial^{a}g_{2}g_{1}\rangle \psi_{2}+\langle k_{\alpha \beta }g_{1}g_{2}\rangle \partial_{\beta }\psi_{2}\right)=0 \end{array},$$

(11) $$\begin{array}{l} \langle c\varrho g_{2}\rangle \left(\dot{\theta }+\langle g_{1}\rangle {\dot{\psi }}_{1}+\langle g_{2}\rangle {\dot{\psi }}_{2}\right)+\langle k_{ab}\partial^{a}g_{2}\rangle \partial_{b}\theta +\langle k_{ab}\partial^{a}g_{1}\partial^{j}g_{2}\rangle \psi_{1}+\langle k_{a\alpha }\partial^{a}g_{2}g_{1}\rangle \partial_{\alpha }\psi_{1}+\\ +\langle k_{ab}\partial^{a}g_{2}\partial^{b}g_{2}\rangle \psi_{2}+\langle k_{a\alpha }\partial^{a}g_{2}g_{2}\rangle \partial_{\alpha }\psi_{2}-\partial_{\alpha }\left(\langle k_{a\alpha }g_{2}\rangle \partial_{a}\theta +\langle k_{a\alpha }\partial^{a}g_{1}g_{2}\rangle \psi_{1}\right)+\\ -\partial_{\alpha }\left(\langle k_{\alpha \beta }g_{1}g_{2}\rangle \partial_{\beta }\psi_{1}+\langle k_{a\alpha }\partial^{a}g_{2}g_{2}\rangle \psi_{2}+\langle k_{\alpha \beta }g_{2}g_{2}\rangle \partial_{\beta }\psi_{2}\right)=0 \end{array}.$$

To prepare an algorithm of the finite difference method solving equations of the tolerance model, some assumptions had to be made at this stage. It was assumed that a non-stationary, two-dimensional problem would be considered. The character of the fluctuation shape functions, adopted and shown in the Figure 2, was also considered, which led to modified equations:

(12) $$\langle c\varrho \rangle \dot{\theta }-\partial_{1}\left(\langle k_{11}\rangle \partial_{1}\theta +\langle k_{11}\partial^{1}g_{1}\rangle \psi_{1}\right)-\partial_{2}\left(\langle k_{22}\rangle \partial_{2}\theta +\langle k_{22}\partial^{2}g_{2}\rangle \psi_{2}\right)=0,$$

(13) $$\langle c\varrho g_{1}g_{1}\rangle {\dot{\psi }}_{1}+\langle k_{11}\partial^{1}g_{1}\rangle \partial_{1}\theta +\langle k_{11}\partial^{1}g_{1}\partial^{1}g_{1}\rangle \psi_{1}+\langle k_{22}\partial^{2}g_{1}\partial^{2}g_{1}\rangle \psi_{1}=0,$$

(14) $$\langle c\varrho g_{2}g_{2}\rangle {\dot{\psi }}_{2}+\langle k_{22}\partial^{2}g_{2}\rangle \partial_{2}\theta +\langle k_{11}\partial^{1}g_{2}\partial^{1}g_{2}\rangle \psi_{2}+\langle k_{22}\partial^{2}g_{2}\partial^{2}g_{2}\rangle \psi_{2}=0.$$

**Figure 3:**
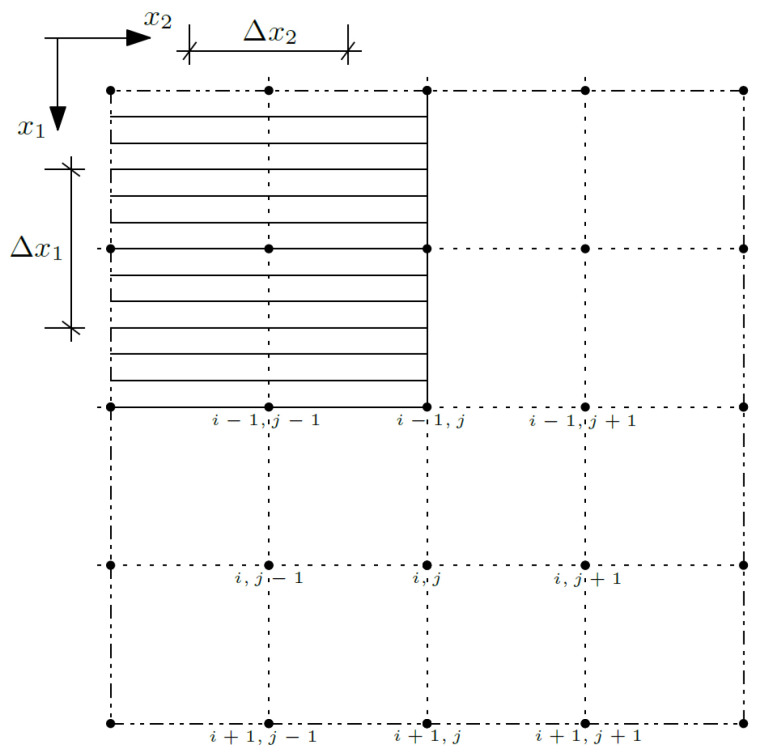
Discretization of the composite.

In the next step, Equations (12)–(14) was reformulated using the differential quotient formulas and written for the node (*i*,*j*):

(15) $$\begin{array}{l} \partial_{1}\langle k_{11}\rangle \frac{\theta_{\left(m\right)}^{i+1,j}-\theta_{\left(m\right)}^{i-1,j}}{2\Delta x_{1}}+\langle k_{11}\rangle \frac{\theta_{\left(m\right)}^{i+1,j}-2\theta_{\left(m\right)}^{i,j}+\theta_{\left(m\right)}^{i-1,j}}{{\left(\Delta x_{1}\right)}^{2}}+\partial_{1}\langle k_{11}\partial^{1}g_{1}\rangle \psi_{1(m)}^{i,j}+\\ +\langle k_{11}\partial^{1}g_{1}\rangle \frac{\psi_{1(m)}^{i+1,j}-\psi_{1(m)}^{i-1,j}}{2\Delta x_{1}}+\partial_{2}\langle k_{22}\rangle \frac{\theta_{\left(m\right)}^{i,j+1}-\theta_{\left(m\right)}^{i,j-1}}{2\Delta x_{2}}+\langle k_{22}\rangle \frac{\theta_{\left(m\right)}^{i,j+1}-2\theta_{\left(m\right)}^{i,j}+\theta_{\left(m\right)}^{i,j-1}}{{\left(\Delta x_{1}\right)}^{2}}+\\ +\partial_{2}\langle k_{22}\partial^{2}g_{2}\rangle \psi_{2(m)}^{i,j}+\langle k_{22}\partial^{2}g_{2}\rangle \frac{\psi_{2(m)}^{i,j+1}-\psi_{2(m)}^{i,j-1}}{2\Delta x_{2}}=\langle c\varrho \rangle \frac{\theta_{\left(m+1\right)}^{i,j}-\theta_{\left(m\right)}^{i,j}}{\Delta t} \end{array},$$

(16) $$\langle k_{11}\partial^{1}g_{1}\rangle \frac{\theta_{\left(m\right)}^{i+1,j}-\theta_{\left(m\right)}^{i-1,j}}{2\Delta x_{1}}+\langle k_{11}\partial^{1}g_{1}\partial^{1}g_{1}\rangle \psi_{1(m)}^{i,j}+\langle k_{22}\partial^{2}g_{1}\partial^{2}g_{1}\rangle \psi_{1(m)}^{i,j}=-\langle c\varrho g_{1}g_{1}\rangle \frac{\psi_{1(m+1)}^{i,j}-\psi_{1(m)}^{i,j}}{\Delta t},$$

(17) $$\langle k_{22}\partial^{2}g_{2}\rangle \frac{\theta_{\left(m\right)}^{i,j+1}-\theta_{\left(m\right)}^{i,j-1}}{2\Delta x_{2}}+\langle k_{11}\partial^{1}g_{2}\partial^{1}g_{2}\rangle \psi_{2(m)}^{i,j}+\langle k_{22}\partial^{2}g_{2}\partial^{2}g_{2}\rangle \psi_{2(m)}^{i,j}=-\langle c\varrho g_{2}g_{2}\rangle \frac{\psi_{2(m+1)}^{i,j}-\psi_{2(m)}^{i,j}}{\Delta t}.$$

where Δ*x*_1_ and Δ*x*_2_ define the distances between nodes in directions *x*_1_ (*i*) and *x*_2_ (*j*); confer Figure 3. By *t* the time coordinate is denoted, and by *m*, the time steps.

The nodes were introduced to the center of each subcell and the interfaces between the cells and between the subcells, so the number of the nodes *m*_1_ in direction *x*_1_ is equal to 4*N*_1_ + 1, where *N*_1_ is a number of the cells in direction *x*_1_, and analogously, the number of the nodes *m*_2_ in direction *x*_2_ is equal to 4*N*_2_ + 1, where *N*_2_ is a number of the cells in direction *x*_2_.

Next, the boundary conditions have to be specified, as they affect the process of the algorithm (if we know the values of the desired unknown at the selected nodes, we do not write the equation associated with that unknown at those nodes).

### 3.1. Matrix of Coefficients

The creation of the algorithm was started by building a matrix of coefficients found in the equations.

#### 3.1.1. Equation for Macrotemperature θ

**Figure 4:**
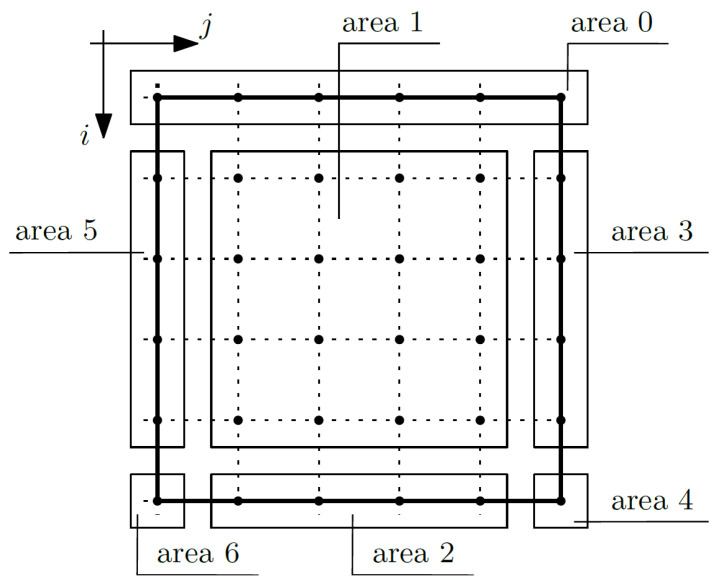
Areas of the composite—macrotemperature θ.

Equation (15) is related to the macrotemperature θ. Considering this equation, the composite nodes were grouped into areas; confer Figure 4.

It was assumed that the total temperature will be known at the nodes of area 0 (for *i* equals 1 and *j* from 1 to *m*_1_), which translates to the fact that the macrotemperature will also be known, and we do not write Equation (15) for these nodes.

The nodes of area 1 (for *i* from 2 to *m*_1_ − 1 and for *j* from 2 to *m*_2_ − 1) are internal nodes and are not affected by the boundary conditions, and it is necessary to write Equation (15) for these nodes. By writing an equation for a given node, the coefficients for the unknown at that node (*i*, *j*), but also at the node above it (*i* − 1, *j*), below it (*i* + 1, *j*), to the right (*i*, *j* + 1), and to the left (*i*, *j* − 1), were grouped. For the purposes of this work, simplified nomenclature was adopted for the coefficient groups. The coefficients for the unknown (macrotemperature θ) in the node (*i*, *j*) in this area are written below:

(18) $$coeff_{i,j}\theta =-\frac{2}{{\left(\Delta x_{1}\right)}^{2}}\langle k_{11}\rangle -\frac{2}{{\left(\Delta x_{2}\right)}^{2}}\langle k_{22}\rangle ,$$

the coefficients in the node (*i* − 1, *j*):

(19) $$coeff_{i-1,j}\theta =\frac{1}{{\left(\Delta x_{1}\right)}^{2}}\langle k_{11}\rangle -\frac{1}{2\Delta x_{1}}\partial_{1}\langle k_{11}\rangle ,$$

the coefficients in the node (*i* + 1, *j*):

(20) $$coeff_{i+1,j}\theta =\frac{1}{{\left(\Delta x_{1}\right)}^{2}}\langle k_{11}\rangle +\frac{1}{2\Delta x_{1}}\partial_{1}\langle k_{11}\rangle ,$$

the coefficients in the node (*i*, *j* − 1):

(21) $$coeff_{i,j-1}\theta =\frac{1}{{\left(\Delta x_{2}\right)}^{2}}\langle k_{22}\rangle -\frac{1}{2\Delta x_{2}}\partial_{2}\langle k_{22}\rangle ,$$

the coefficients in the node (*i*, *j* + 1):

(22) $$coeff_{i,j+1}\theta =\frac{1}{{\left(\Delta x_{2}\right)}^{2}}\langle k_{22}\rangle +\frac{1}{2\Delta x_{2}}\partial_{2}\langle k_{22}\rangle .$$

The nodes of area 2 (for *i* equal to *m*_1_ and for *j* from 2 to *m*_2_ − 1) are the nodes on the bottom surface of the composite. On this surface, the Robin boundary conditions are assumed. These conditions are related to a heat exchange and are expressed according to the formula:

(23) $$\langle k_{11}\rangle \partial_{1}\Theta =-U_{1}\left(\Theta -\Theta_{e}\right),$$

where *U*_1_ is a heat transfer coefficient and Θ*_e_* is an external temperature. This condition was written by using the micro-macro decomposition assumption and the differential quotient formulas:

(24) $$\begin{array}{l} \frac{\theta^{i+1,j}-\theta^{i-1,j}}{2\Delta x_{1}}\left[{\langle k_{11}\rangle }_{2}-\frac{{\langle k_{11}g_{1}\rangle }_{2}\left({\langle k_{11}\partial^{1}g_{1}\rangle }_{2}+{\langle U_{1}g_{1}\rangle }_{2}\right)}{{\langle k_{11}g_{1}\partial^{1}g_{1}\rangle }_{2}+{\langle U_{1}g_{1}g_{1}\rangle }_{2}}\right]+\\ +\theta^{i,j}\left[{\langle U_{1}\rangle }_{2}-\frac{{\langle U_{1}g_{1}\rangle }_{2}\left({\langle k_{11}\partial^{1}g_{1}\rangle }_{2}+{\langle U_{1}g_{1}\rangle }_{2}\right)}{{\langle k_{11}g_{1}\partial^{1}g_{1}\rangle }_{2}+{\langle U_{1}g_{1}g_{1}\rangle }_{2}}\right]=\\ ={\langle U_{1}\Theta_{e}\rangle }_{2}-\frac{{\langle U_{1}\Theta_{e}g_{1}\rangle }_{2}\left({\langle k_{11}\partial^{1}g_{1}\rangle }_{2}+{\langle U_{1}g_{1}\rangle }_{2}\right)}{{\langle k_{11}g_{1}\partial^{1}g_{1}\rangle }_{2}+{\langle U_{1}g_{1}g_{1}\rangle }_{2}} \end{array},$$

where the averaging operation is valid only in direction *x*_2_. From the above equation, the component θ*^i^*^+1,*j*^ was determined, which allowed the elimination of the unknown in the virtual node (*i* + 1, *j*) from Equation (15). The formulas describing the individual coefficients are shown below:

(25) $$\begin{array}{l} coeff_{i,j}\theta =-2\left(\frac{{\langle k_{11}\rangle }_{1}}{{\left(\Delta x_{1}\right)}^{2}}+\frac{{\langle k_{22}\rangle }_{1}}{{\left(\Delta x_{2}\right)}^{2}}\right)+\\ -\left(\partial_{1}{\langle k_{11}\rangle }_{2}+\frac{2{\langle k_{11}\rangle }_{2}}{\Delta x_{1}}\right)\frac{{\langle U_{1}\rangle }_{2}\left({\langle k_{11}g_{1}\partial^{1}g_{1}\rangle }_{2}+{\langle U_{1}g_{1}g_{1}\rangle }_{2}\right)-{\langle U_{1}g_{1}\rangle }_{2}\left({\langle k_{11}\partial^{1}g_{1}\rangle }_{2}+{\langle U_{1}g_{1}\rangle }_{2}\right)}{{\langle k_{11}\rangle }_{2}\left({\langle k_{11}g_{1}\partial^{1}g_{1}\rangle }_{2}+{\langle U_{1}g_{1}g_{1}\rangle }_{2}\right)-{\langle k_{11}g_{1}\rangle }_{2}\left({\langle k_{11}\partial^{1}g_{1}\rangle }_{2}+{\langle U_{1}g_{1}\rangle }_{2}\right)} \end{array},$$

(26) $$coeff_{i-1,j}\theta =\frac{2}{{\left(\Delta x_{1}\right)}^{2}}{\langle k_{11}\rangle }_{2},$$

(27) $$coeff_{i,j-1}\theta =\frac{1}{{\left(\Delta x_{2}\right)}^{2}}{\langle k_{22}\rangle }_{2}-\frac{1}{2\Delta x_{2}}\partial_{2}{\langle k_{22}\rangle }_{2},$$

(28) $$coeff_{i,j+1}\theta =\frac{1}{{\left(\Delta x_{2}\right)}^{2}}{\langle k_{22}\rangle }_{2}+\frac{1}{2\Delta x_{2}}\partial_{2}{\langle k_{22}\rangle }_{2}.$$

The nodes of area 3 (for *i* from 2 to *m*_1_ − 1 and for *j* equals *m*_2_) represent the nodes on the right surface of the composite. On this surface, the Robin boundary conditions are also assumed. These conditions are expressed according to the formula:

(29) $$\langle k_{22}\rangle \partial_{2}\Theta =-U_{2}\left(\Theta -\Theta_{e}\right),$$

where *U*_2_ is a heat transfer coefficient. This condition was written by using the micro-macro decomposition assumption and the differential quotient formulas:

(30) $$\begin{array}{l} \frac{\theta^{i,j+1}-\theta^{i,j-1}}{2\Delta x_{2}}\left[{\langle k_{22}\rangle }_{1}-\frac{{\langle k_{22}g_{2}\rangle }_{1}\left({\langle k_{22}\partial^{2}g_{2}\rangle }_{1}+{\langle U_{2}g_{2}\rangle }_{1}\right)}{{\langle k_{22}g_{2}\partial^{2}g_{2}\rangle }_{1}+{\langle U_{2}g_{2}g_{2}\rangle }_{1}}\right]+\\ +\theta^{i,j}\left[{\langle U_{2}\rangle }_{1}-\frac{{\langle U_{2}g_{2}\rangle }_{1}\left({\langle k_{22}\partial^{2}g_{2}\rangle }_{1}+{\langle U_{2}g_{2}\rangle }_{1}\right)}{{\langle k_{22}g_{2}\partial^{2}g_{2}\rangle }_{1}+{\langle U_{2}g_{2}g_{2}\rangle }_{1}}\right]=\\ ={\langle U_{2}\Theta_{e}\rangle }_{1}-\frac{{\langle U_{2}\Theta_{e}g_{2}\rangle }_{1}\left({\langle k_{22}\partial^{2}g_{2}\rangle }_{1}+{\langle U_{2}g_{2}\rangle }_{1}\right)}{{\langle k_{22}g_{2}\partial^{2}g_{2}\rangle }_{1}+{\langle U_{2}g_{2}g_{2}\rangle }_{1}} \end{array},$$

where the averaging operation is valid only in direction *x*_1_. From the above equation, the component θ*^i,j+^*^1^ was determined, which allowed the elimination of the unknown in the virtual node (*i*, *j* + 1) from Equation (15). The formulas describing the individual coefficients are shown below:

(31) $$\begin{array}{l} coeff_{i,j}\theta =-2\left(\frac{{\langle k_{11}\rangle }_{1}}{{\left(\Delta x_{1}\right)}^{2}}+\frac{{\langle k_{22}\rangle }_{1}}{{\left(\Delta x_{2}\right)}^{2}}\right)+\\ -\left(\partial_{2}{\langle k_{22}\rangle }_{1}+\frac{2{\langle k_{22}\rangle }_{1}}{\Delta x_{2}}\right)\frac{{\langle U_{2}\rangle }_{1}\left({\langle k_{22}g_{2}\partial^{2}g_{2}\rangle }_{1}+{\langle U_{2}g_{2}g_{2}\rangle }_{1}\right)-{\langle U_{2}g_{2}\rangle }_{1}\left({\langle k_{22}\partial^{2}g_{2}\rangle }_{1}+{\langle U_{2}g_{2}\rangle }_{1}\right)}{{\langle k_{22}\rangle }_{1}\left({\langle k_{22}g_{2}\partial^{2}g_{2}\rangle }_{1}+{\langle U_{2}g_{2}g_{2}\rangle }_{1}\right)-{\langle k_{22}g_{2}\rangle }_{1}\left({\langle k_{22}\partial^{2}g_{2}\rangle }_{1}+{\langle U_{2}g_{2}\rangle }_{1}\right)} \end{array},$$

(32) $$coeff_{i+1,j}\theta =\frac{1}{{\left(\Delta x_{1}\right)}^{2}}{\langle k_{11}\rangle }_{1}+\frac{1}{2\Delta x_{1}}\partial_{1}{\langle k_{11}\rangle }_{1},$$

(33) $$coeff_{i-1,j}\theta =\frac{1}{{\left(\Delta x_{1}\right)}^{2}}{\langle k_{11}\rangle }_{1}-\frac{1}{2\Delta x_{1}}\partial_{1}{\langle k_{11}\rangle }_{1},$$

(34) $$coeff_{i,j-1}\theta =\frac{2}{{\left(\Delta x_{2}\right)}^{2}}{\langle k_{22}\rangle }_{1}.$$

The node of area 4 (for *i* equals *m*_1_ and for *j* equal to *m*_2_) is the node in the bottom right corner of the composite. In this area, the boundary conditions described by Equations (23) and (29) were considered. Therefore, the formulas describing the coefficients are as follows:

(35) $$\begin{array}{l} coeff_{i,j}\theta =-2\left(\frac{{\langle k_{11}\rangle }_{1}}{{\left(\Delta x_{1}\right)}^{2}}+\frac{{\langle k_{22}\rangle }_{1}}{{\left(\Delta x_{2}\right)}^{2}}\right)+\\ -\left(\partial_{1}{\langle k_{11}\rangle }_{2}+\frac{2{\langle k_{11}\rangle }_{2}}{\Delta x_{1}}\right)\frac{{\langle U_{1}\rangle }_{2}\left({\langle k_{11}g_{1}\partial^{1}g_{1}\rangle }_{2}+{\langle U_{1}g_{1}g_{1}\rangle }_{2}\right)-{\langle U_{1}g_{1}\rangle }_{2}\left({\langle k_{11}\partial^{1}g_{1}\rangle }_{2}+{\langle U_{1}g_{1}\rangle }_{2}\right)}{{\langle k_{11}\rangle }_{2}\left({\langle k_{11}g_{1}\partial^{1}g_{1}\rangle }_{2}+{\langle U_{1}g_{1}g_{1}\rangle }_{2}\right)-{\langle k_{11}g_{1}\rangle }_{2}\left({\langle k_{11}\partial^{1}g_{1}\rangle }_{2}+{\langle U_{1}g_{1}\rangle }_{2}\right)}+\\ -\left(\partial_{2}{\langle k_{22}\rangle }_{1}+\frac{2{\langle k_{22}\rangle }_{1}}{\Delta x_{2}}\right)\frac{{\langle U_{2}\rangle }_{1}\left({\langle k_{22}g_{2}\partial^{2}g_{2}\rangle }_{1}+{\langle U_{2}g_{2}g_{2}\rangle }_{1}\right)-{\langle U_{2}g_{2}\rangle }_{1}\left({\langle k_{22}\partial^{2}g_{2}\rangle }_{1}+{\langle U_{2}g_{2}\rangle }_{1}\right)}{{\langle k_{22}\rangle }_{1}\left({\langle k_{22}g_{2}\partial^{2}g_{2}\rangle }_{1}+{\langle U_{2}g_{2}g_{2}\rangle }_{1}\right)-{\langle k_{22}g_{2}\rangle }_{1}\left({\langle k_{22}\partial^{2}g_{2}\rangle }_{1}+{\langle U_{2}g_{2}\rangle }_{1}\right)} \end{array},$$

(36) $$coeff_{i-1,j}\theta =\frac{2}{{\left(\Delta x_{1}\right)}^{2}}{\langle k_{11}\rangle }_{2},$$

(37) $$coeff_{i,j-1}\theta =\frac{2}{{\left(\Delta x_{2}\right)}^{2}}{\langle k_{22}\rangle }_{2}.$$

The nodes of area 5 (for *i* from 2 to *m*_1_ − 1 and for *j* equal to 1) represent the nodes on the left surface of the composite. It was assumed that this edge is thermally insulated:

(38) $$\langle k_{22}\rangle \partial_{2}\Theta =0,$$

which leads to the condition:

(39) $$\partial_{2}\theta =0\to \theta^{i,j+1}=\theta^{i,j-1},$$

and allows to the elimination of the unknown in the virtual nodes (*i*, *j* − 1) from the equation. The formulas describing the individual coefficients are shown below:

(40) $$coeff_{i,j}\theta =-\frac{2}{{\left(\Delta x_{1}\right)}^{2}}{\langle k_{11}\rangle }_{1}-\frac{2}{{\left(\Delta x_{2}\right)}^{2}}{\langle k_{22}\rangle }_{1},$$

(41) $$coeff_{i+1,j}\theta =\frac{1}{{\left(\Delta x_{1}\right)}^{2}}{\langle k_{11}\rangle }_{1}+\frac{1}{2\Delta x_{1}}\partial_{1}{\langle k_{11}\rangle }_{1},$$

(42) $$coeff_{i-1,j}\theta =\frac{1}{{\left(\Delta x_{1}\right)}^{2}}{\langle k_{11}\rangle }_{1}-\frac{1}{2\Delta x_{1}}\partial_{1}{\langle k_{11}\rangle }_{1},$$

(43) $$coeff_{i,j+1}\theta =\frac{2}{{\left(\Delta x_{2}\right)}^{2}}{\langle k_{22}\rangle }_{1}.$$

where the averaging operation is valid only along *x*_1_.

The node of area 6 (for *i* equals *m*_1_ and for *j* equal to 1) is the node in the bottom left corner of the composite. In this area, the boundary conditions described by Equations (23) and (21) are considered. Therefore, the formulas describing the coefficients are as follows:

(44) $$\begin{array}{l} coeff_{i,j}\theta =-2\left(\frac{{\langle k_{11}\rangle }_{1}}{{\left(\Delta x_{1}\right)}^{2}}+\frac{{\langle k_{22}\rangle }_{1}}{{\left(\Delta x_{2}\right)}^{2}}\right)+\\ -\left(\partial_{1}{\langle k_{11}\rangle }_{2}+\frac{2{\langle k_{11}\rangle }_{2}}{\Delta x_{1}}\right)\frac{{\langle U_{1}\rangle }_{2}\left({\langle k_{11}g_{1}\partial^{1}g_{1}\rangle }_{2}+{\langle U_{1}g_{1}g_{1}\rangle }_{2}\right)-{\langle U_{1}g_{1}\rangle }_{2}\left({\langle k_{11}\partial^{1}g_{1}\rangle }_{2}+{\langle U_{1}g_{1}\rangle }_{2}\right)}{{\langle k_{11}\rangle }_{2}\left({\langle k_{11}g_{1}\partial^{1}g_{1}\rangle }_{2}+{\langle U_{1}g_{1}g_{1}\rangle }_{2}\right)-{\langle k_{11}g_{1}\rangle }_{2}\left({\langle k_{11}\partial^{1}g_{1}\rangle }_{2}+{\langle U_{1}g_{1}\rangle }_{2}\right)} \end{array},$$

(45) $$coeff_{i-1,j}\theta =\frac{2}{{\left(\Delta x_{1}\right)}^{2}}{\langle k_{11}\rangle }_{2},$$

(46) $$coeff_{i,j+1}\theta =\frac{2}{{\left(\Delta x_{2}\right)}^{2}}{\langle k_{22}\rangle }_{2}.$$

where the averaging operation is valid only along *x*_1_.

In each area, in the case of the macrotemperature θ, in the node (*i*, *j*), the coefficient related to the time coordinate was added:

(47) $$coeff_{i,j}\theta_{t}=-\frac{1}{\Delta t}\langle c\varrho \rangle ,$$

and it was assumed that in the initial time the values of all unknowns are known.

There are also the fluctuation amplitudes of the temperature ψ_1_ and ψ_2_ as unknowns in Equation (15). The coefficients for the fluctuation amplitudes ψ_1_ are as follows:

- area 1,

(48) $$coeff_{i,j}\psi_{1}=\partial_{1}\langle k_{11}\partial^{1}g_{1}\rangle ,$$

(49) $$coeff_{i-1,j}\psi_{1}=-\frac{1}{2\Delta x_{1}}\langle k_{11}\partial^{1}g_{1}\rangle ,$$

(50) $$coeff_{i+1,j}\psi_{1}=\frac{1}{2\Delta x_{1}}\langle k_{11}\partial^{1}g_{1}\rangle ,$$

- areas 2, 4, 6,

(51) $$coeff_{i,j}\psi_{1}=\partial_{1}{\langle k_{11}\partial^{1}g_{1}\rangle }_{2}+\frac{1}{\Delta x_{1}}{\langle k_{11}\partial^{1}g_{1}\rangle }_{2},$$

(52) $$coeff_{i-1,j}\psi_{1}=-\frac{1}{\Delta x_{1}}{\langle k_{11}\partial^{1}g_{1}\rangle }_{2},$$

- area 3,

(53) $$coeff_{i,j}\psi_{1}=\partial_{1}{\langle k_{11}\partial^{1}g_{1}\rangle }_{1},$$

(54) $$coeff_{i-1,j}\psi_{1}=-\frac{1}{2\Delta x_{1}}{\langle k_{11}\partial^{1}g_{1}\rangle }_{1},$$

(55) $$coeff_{i+1,j}\psi_{1}=\frac{1}{2\Delta x_{1}}{\langle k_{11}\partial^{1}g_{1}\rangle }_{1},$$

- area 5,

(56) $$coeff_{i,j}\psi_{1}=\partial_{1}{\langle k_{11}\partial^{1}g_{1}\rangle }_{1},$$

(57) $$coeff_{i-1,j}\psi_{1}=-\frac{1}{2\Delta x_{1}}{\langle k_{11}\partial^{1}g_{1}\rangle }_{1},$$

(58) $$coeff_{i+1,j}\psi_{1}=\frac{1}{2\Delta x_{1}}{\langle k_{11}\partial^{1}g_{1}\rangle }_{1}.$$

The coefficients for the fluctuation amplitudes ψ_2_ are as follows:

- area 1,

(59) $$coeff_{i,j}\psi_{2}=\partial_{2}\langle k_{22}\partial^{2}g_{2}\rangle ,$$

(60) $$coeff_{i,j-1}\psi_{2}=-\frac{1}{2\Delta x_{2}}\langle k_{22}\partial^{2}g_{2}\rangle ,$$

(61) $$coeff_{i,j+1}\psi_{2}=\frac{1}{2\Delta x_{2}}\langle k_{22}\partial^{2}g_{2}\rangle ,$$

- areas 2,

(62) $$coeff_{i,j}\psi_{2}=\partial_{2}{\langle k_{22}\partial^{2}g_{2}\rangle }_{2},$$

(63) $$coeff_{i,j-1}\psi_{2}=-\frac{1}{2\Delta x_{2}}{\langle k_{22}\partial^{2}g_{2}\rangle }_{2},$$

(64) $$coeff_{i,j+1}\psi_{2}=\frac{1}{2\Delta x_{2}}{\langle k_{22}\partial^{2}g_{2}\rangle }_{2},$$

- areas 3, 4

(65) $$coeff_{i,j}\psi_{2}=\partial_{2}{\langle k_{22}\partial^{2}g_{2}\rangle }_{1}+\frac{1}{\Delta x_{2}}{\langle k_{22}\partial^{2}g_{2}\rangle }_{1},$$

(66) $$coeff_{i,j-1}\psi_{2}=-\frac{1}{\Delta x_{2}}{\langle k_{22}\partial^{2}g_{2}\rangle }_{1},$$

- areas 5, 6

(67) $$coeff_{i,j}\psi_{2}=\partial_{2}{\langle k_{22}\partial^{2}g_{2}\rangle }_{1},$$

(68) $$coeff_{i,j+1}\psi_{2}=\partial_{2}{\langle k_{22}\partial^{2}g_{2}\rangle }_{1}-\frac{1}{\Delta x_{2}}{\langle k_{22}\partial^{2}g_{2}\rangle }_{1}.$$

#### 3.1.2. Equation for Fluctuation Amplitude of the Temperature ψ_1_

**Figure 5:**
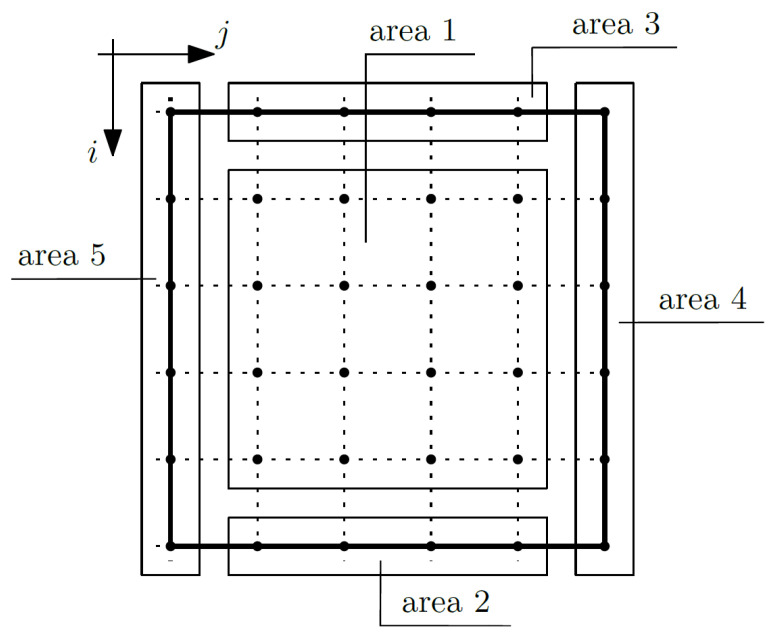
Areas of the composite—fluctuation amplitudes of the temperature ψ_1_.

Equation (16) is related to the fluctuation amplitudes of the temperature ψ_1_. Considering this equation, the composite nodes were grouped into areas; confer Figure 5.

It is assumed that the fluctuation amplitudes of the temperature ψ_1_ will be known for the nodes of area 4 (for *i* from 1 to *m*_1_ and *j* equal to *m*_2_) and area 5 (for *i* from 1 to *m*_1_ and *j* equals 1), which translates to the fact that fluctuation amplitudes of the temperature ψ_1_ are known, and we did not write Equation (16) for these nodes. Analogously, as in the case of Equation (15), the coefficients were grouped and written separately for each unknown. First, the coefficients on the macrotemperature were written as:

- area 1,

(69) $$coeff_{i-1,j}\theta =-\frac{1}{2\Delta x_{1}}\langle k_{11}\partial^{1}g_{1}\rangle ,$$

(70) $$coeff_{i+1,j}\theta =\frac{1}{2\Delta x_{1}}\langle k_{11}\partial^{1}g_{1}\rangle ,$$

- area 2,

(71) $$coeff_{i,j}\theta =\frac{1}{\Delta x_{1}}{\langle k_{11}\partial^{1}g_{1}\rangle }_{2},$$

(72) $$coeff_{i-1,j}\theta =-\frac{1}{\Delta x_{1}}{\langle k_{11}\partial^{1}g_{1}\rangle }_{2},$$

- area 3,

(73) $$coeff_{i,j}\theta =-\frac{1}{\Delta x_{1}}{\langle k_{11}\partial^{1}g_{1}\rangle }_{2},$$

(74) $$coeff_{i+1,j}\theta =\frac{1}{\Delta x_{1}}{\langle k_{11}\partial^{1}g_{1}\rangle }_{2}.$$

Then, the coefficients on the fluctuation amplitudes of the temperature were written:

- area 1,

(75) $$coeff_{i-1,j}\psi_{1}=\langle k_{11}\partial^{1}g_{1}\partial^{1}g_{1}\rangle +\langle k_{22}\partial^{2}g_{1}\partial^{2}g_{1}\rangle ,$$

- areas 2,

(76) $$coeff_{i,j}\psi_{1}=\langle k_{11}\partial^{1}g_{1}\partial^{1}g_{1}\rangle +\langle k_{22}\partial^{2}g_{1}\partial^{2}g_{1}\rangle ,$$

- area 3,

(77) $$coeff_{i,j}\psi_{1}=\langle k_{11}\partial^{1}g_{1}\partial^{1}g_{1}\rangle +\langle k_{22}\partial^{2}g_{1}\partial^{2}g_{1}\rangle .$$

In each area, in the case of the fluctuation amplitudes of the temperature ψ_1_, in the node (*i*, *j*), the coefficient related to the time coordinate was added:

(78) $$coeff_{i,j}\psi_{1t}=-\frac{1}{\Delta t}\langle c\varrho g_{1}g_{1}\rangle .$$

#### 3.1.3. Equation for Fluctuation Amplitude of the Temperature ψ_2_

**Figure 6:**
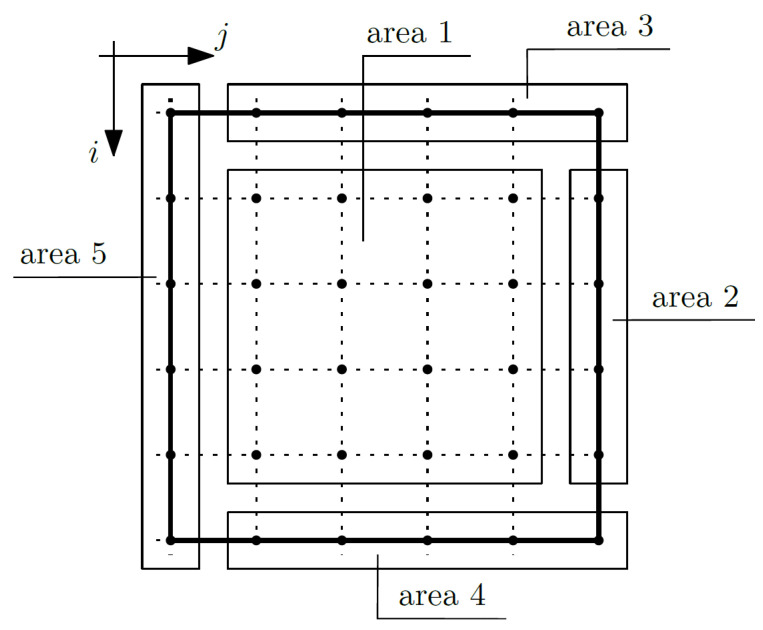
Areas of the composite—fluctuation amplitudes of the temperature ψ_2_.

Equation (17) is related to the fluctuation amplitudes of the temperature ψ_2_(*x*_1_, *x*_2_). Considering this equation, the composite nodes were grouped into areas again; confer Figure 6.

It is assumed that the fluctuation amplitudes of the temperature ψ_2_ will be known for the nodes of area 3 (for *i* equal to 1 and *j* from 2 to *m*_2_), area 4 (for *i* equals *m*_1_ and *j* from 2 to *m*_2_), and area 5 (for *i* from 1 to *m*_1_ and *j* equals 1), which translates to the fact that fluctuation amplitudes of the temperature ψ_2_ are known, and we did not write Equation (17) for these nodes. First, the coefficients of the macrotemperature were written:

- area 1,

(79) $$coeff_{i,j+1}\theta =\frac{1}{2\Delta x_{2}}\langle k_{22}\partial^{2}g_{2}\rangle ,$$

(80) $$coeff_{i,j-1}\theta =-\frac{1}{2\Delta x_{2}}\langle k_{22}\partial^{2}g_{2}\rangle ,$$

- area 2,

(81) $$coeff_{i,j}\theta =\frac{1}{\Delta x_{2}}{\langle k_{22}\partial^{2}g_{2}\rangle }_{1},$$

(82) $$coeff_{i-1,j}\theta =-\frac{1}{\Delta x_{2}}{\langle k_{22}\partial^{2}g_{2}\rangle }_{1}.$$

Then, the coefficients on the fluctuation amplitudes of the temperature were written:

- area 1,

(83) $$coeff_{i,j}\psi_{2}=\langle k_{22}\partial^{1}g_{2}\partial^{1}g_{2}\rangle +\langle k_{22}\partial^{2}g_{2}\partial^{2}g_{2}\rangle ,$$

- area 2,

(84) $$coeff_{i,j}\psi_{2}={\langle k_{22}\partial^{1}g_{2}\partial^{1}g_{2}\rangle }_{1}+{\langle k_{22}\partial^{2}g_{2}\partial^{2}g_{2}\rangle }_{1}.$$

In each area, in the case of the fluctuation amplitude of the temperature ψ_2_, in the node (*i*, *j*), the coefficient related to the time coordinate was added:

(85) $$coeff_{i,j}\psi_{2t}=-\frac{1}{\Delta t}\langle c\varrho g_{2}g_{2}\rangle .$$

### 3.2. Vector of Free Terms

**Figure 7:**
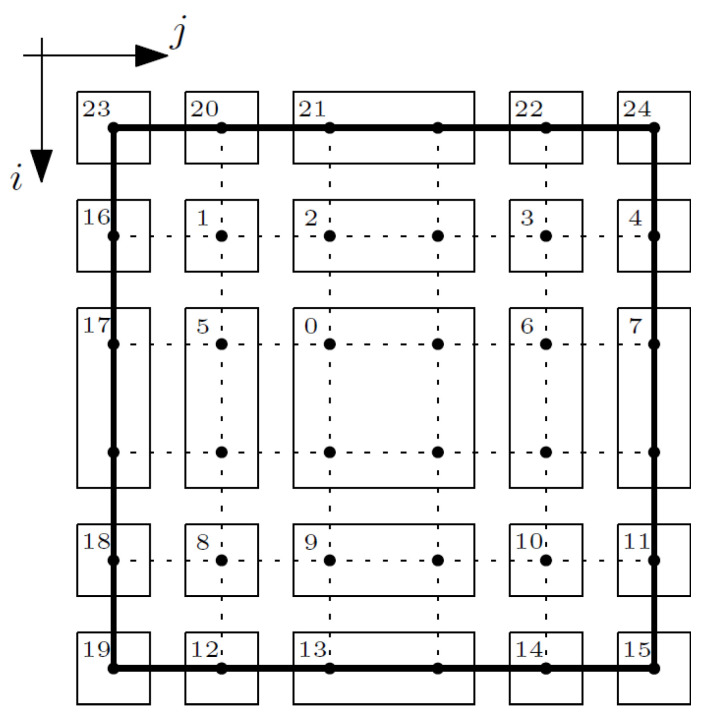
Areas of the composite—vector of free terms.

In the case of the free terms vector, the procedure is analogous to the matrix of coefficients. The composite nodes were grouped into areas; confer Figure 7.

The vector of free terms is connected with the boundary conditions; therefore, free terms do not appear in equations written for area 0. The free terms do not concern the nodes in areas 23 and 24, because at these nodes the equations are not used. According to the boundary conditions, described above, the values of the macrotemperature θ, are known for the top surface of the composite, so these values occurred as free terms in the individual equations written for the nodes of areas 1–4 and 16 (*i* − 1, *j*) and areas 20–22 (*i*, *j*; *i*, *j* − 1; *i*, *j* + 1).

The values of the fluctuation amplitudes of the temperature ψ_1_ are known for the left and right surfaces of the composite, so these values occurred as free terms in the individual equations written for the nodes of areas 4, 7, 11, and 16–18 (*i*, *j*; *i*−1, *j*; *i* + 1, *j*), for the nodes of areas 15 and 19 (*i*, *j*; *i* − 1, *j*), for the nodes of areas 1, 5, 8, and 12 (i, *j* − 1), and for the nodes of areas 3, 6, 10, and 14 (*i*, *j* + 1).

In turn, the values of the fluctuation amplitudes of the temperature ψ_2_ are known on the left, top, and bottom surfaces of the composite, so these values occur as free terms in the individual equations written for the nodes of areas 12–14, and 20–22 (*i*, *j*; *i*, *j* − 1; *i*, *j* + 1), for the nodes of areas 16–18 (*i*, *j*; *i* + 1, *j*; *i* − 1; *j*), for the node of area 19 (*i*, *j*; *i* − 1, *j*; *i*, *j* + 1), for the node of area 15 (*i*, *j*; *i*, *j* − 1), for the node of area 1 (*i* − 1, *j*; *i*, *j* − 1), for the nodes of area 5 (*i*, *j* − 1), for the node of area 8 (*i*, *j* − 1; *i* + 1, *j*), for the nodes of areas 2–4 (*i* − 1, *j*), and for the nodes of areas 9–11 (*i* + 1, *j*).

Additionally, for Equation (15) and several areas, there are additional components that need to be added to the free terms. These are associated with the equations expressing the boundary conditions:

- areas 4, 7, and 11,

(86) $$Q_{i,j}\theta =-\left(\partial_{2}{\langle k_{22}\rangle }_{1}+\frac{2{\langle k_{22}\rangle }_{1}}{\Delta x_{2}}\right)\frac{{\langle U_{2}\Theta_{e}\rangle }_{1}-\frac{{\langle U_{2}\Theta_{e}g_{2}\rangle }_{1}\left({\langle k_{22}\partial^{2}g_{2}\rangle }_{1}+{\langle U_{2}g_{2}\rangle }_{1}\right)}{{\langle k_{22}g_{2}\partial^{2}g_{2}\rangle }_{1}+{\langle U_{2}g_{2}g_{2}\rangle }_{1}}}{{\langle k_{22}\rangle }_{1}-\frac{{\langle k_{22}g_{2}\rangle }_{1}\left({\langle k_{22}\partial^{2}g_{2}\rangle }_{1}+{\langle U_{2}g_{2}\rangle }_{1}\right)}{{\langle k_{22}g_{2}\partial^{2}g_{2}\rangle }_{1}+{\langle U_{2}g_{2}g_{2}\rangle }_{1}}},$$

- areas 12, 13, 14, and 19,

(87) $$Q_{i,j}\theta =-\left(\partial_{1}{\langle k_{11}\rangle }_{2}+\frac{2{\langle k_{11}\rangle }_{2}}{\Delta x_{1}}\right)\frac{{\langle U_{1}\Theta_{e}\rangle }_{2}-\frac{{\langle U_{1}\Theta_{e}g_{1}\rangle }_{2}\left({\langle k_{11}\partial^{1}g_{1}\rangle }_{2}+{\langle U_{1}g_{1}\rangle }_{2}\right)}{{\langle k_{11}g_{1}\partial^{1}g_{1}\rangle }_{2}+{\langle U_{1}g_{1}g_{1}\rangle }_{2}}}{{\langle k_{11}\rangle }_{2}-\frac{{\langle k_{11}g_{1}\rangle }_{2}\left({\langle k_{11}\partial^{1}g_{1}\rangle }_{2}+{\langle U_{1}g_{1}\rangle }_{2}\right)}{{\langle k_{11}g_{1}\partial^{1}g_{1}\rangle }_{2}+{\langle U_{1}g_{1}g_{1}\rangle }_{2}}},$$

- area 15,

(88) $$\begin{array}{l} Q_{i,j}\theta =-\left(\partial_{2}{\langle k_{22}\rangle }_{1}+\frac{2{\langle k_{22}\rangle }_{1}}{\Delta x_{2}}\right)\frac{{\langle U_{2}\Theta_{e}\rangle }_{1}-\frac{{\langle U_{2}\Theta_{e}g_{2}\rangle }_{1}\left({\langle k_{22}\partial^{2}g_{2}\rangle }_{1}+{\langle U_{2}g_{2}\rangle }_{1}\right)}{{\langle k_{22}g_{2}\partial^{2}g_{2}\rangle }_{1}+{\langle U_{2}g_{2}g_{2}\rangle }_{1}}}{{\langle k_{22}\rangle }_{1}-\frac{{\langle k_{22}g_{2}\rangle }_{1}\left({\langle k_{22}\partial^{2}g_{2}\rangle }_{1}+{\langle U_{2}g_{2}\rangle }_{1}\right)}{{\langle k_{22}g_{2}\partial^{2}g_{2}\rangle }_{1}+{\langle U_{2}g_{2}g_{2}\rangle }_{1}}}+\\ -\left(\partial_{1}{\langle k_{11}\rangle }_{2}+\frac{2{\langle k_{11}\rangle }_{2}}{\Delta x_{1}}\right)\frac{{\langle U_{1}\Theta_{e}\rangle }_{2}-\frac{{\langle U_{1}\Theta_{e}g_{1}\rangle }_{2}\left({\langle k_{11}\partial^{1}g_{1}\rangle }_{2}+{\langle U_{1}g_{1}\rangle }_{2}\right)}{{\langle k_{11}g_{1}\partial^{1}g_{1}\rangle }_{2}+{\langle U_{1}g_{1}g_{1}\rangle }_{2}}}{{\langle k_{11}\rangle }_{2}-\frac{{\langle k_{11}g_{1}\rangle }_{2}\left({\langle k_{11}\partial^{1}g_{1}\rangle }_{2}+{\langle U_{1}g_{1}\rangle }_{2}\right)}{{\langle k_{11}g_{1}\partial^{1}g_{1}\rangle }_{2}+{\langle U_{1}g_{1}g_{1}\rangle }_{2}}} \end{array},$$

### 3.3. Solution

The Crank–Nicolson method was used [29]. The equations, written for the nodes, form a system of non-uniform linear equations that can be written as follows:

(89) $$\left(1-\alpha \right)Kq^{t+1}+\alpha Kq^{t}+K_{t}q^{t+1}+K_{t}q^{t}=\left(1-\alpha \right)Q^{t+1}+\alpha Q^{t},$$

where parameter α is equal to 0.5. The matrix of coefficients is divided into matrix of coefficients dependent on time coordinate **K***_t_* and independent of time coordinate **K**. The free terms vector is denoted by **Q**, and the unknowns’ vector is denoted by **q**. Each equation in Equations (15)–(17) was written in turn for each node. Solving the above system of equations allows us to know the distribution of the sought unknowns—the macrotemperature θ and the fluctuation amplitudes of the temperatures ψ_1_ and ψ_2_. Then, it is possible to know the distribution of the total temperature Θ by using the micro-macrotemperature assumption as shown in Equation (2). 

## 4. An Example of an Application

The problem analyzed using the algorithm described above was a non-stationary, two dimensional problem of a heat conduction issue in a biperiodic composite with dimensions *L*_1_ and *L*_2_ equal to 1 [m]. The following properties were assumed for the first material in the cell: *c*^1^ = 500 [J kg^−1^ K^−1^], ρ^1^ = 7800 [kg m^−3^], *k_ij_*^1^ = 58 [W m^−1^ K^−1^], and for the second material: *c*^2^ = 920 [J kg^−1^ K^−1^], ρ^2^ = 2720 [kg m^−3^], and *k_ij_*^2^ = 200 [W m^−1^ K^−1^]. The calculations were carried out for the number of the cells *N*_1_ = *N*_2_ = 10. In turn, the share of the first material in the cell along both directions *x*_1_ and *x*_2_ was equal to 1/5.

**Figure 8:**
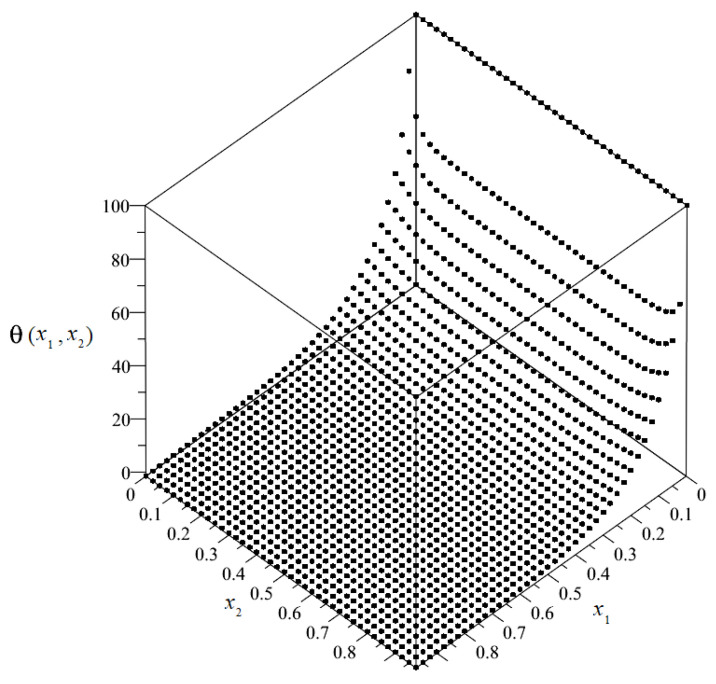
Plot of the distribution of discretized macrotemperature θ.

**Figure 9:**
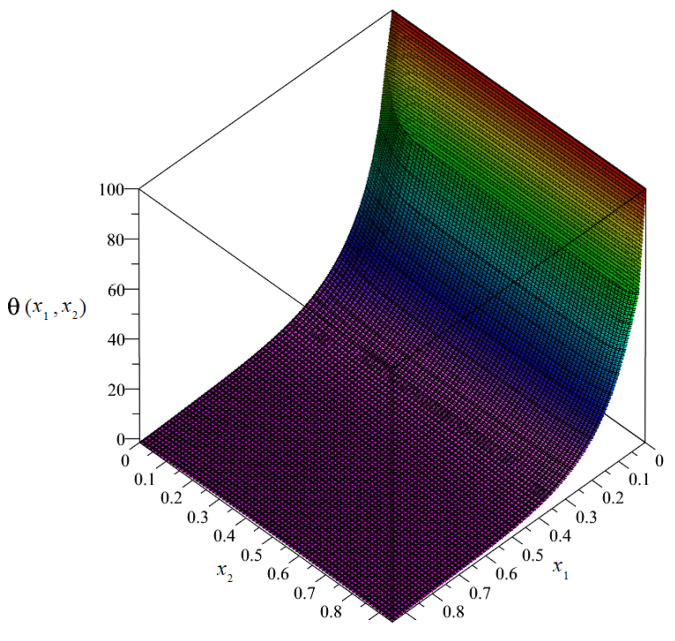
Map of the distribution of macrotemperature θ.

By solving the system of non-uniform Equation (89), it was possible to obtain the plots and the maps of the distributions of the sought unknowns. The plot of the distribution of discretized macrotemperature θ = θ(*x*_1_, *x*_2_) is shown in Figure 8, and as a map in Figure 9.

**Figure 10:**
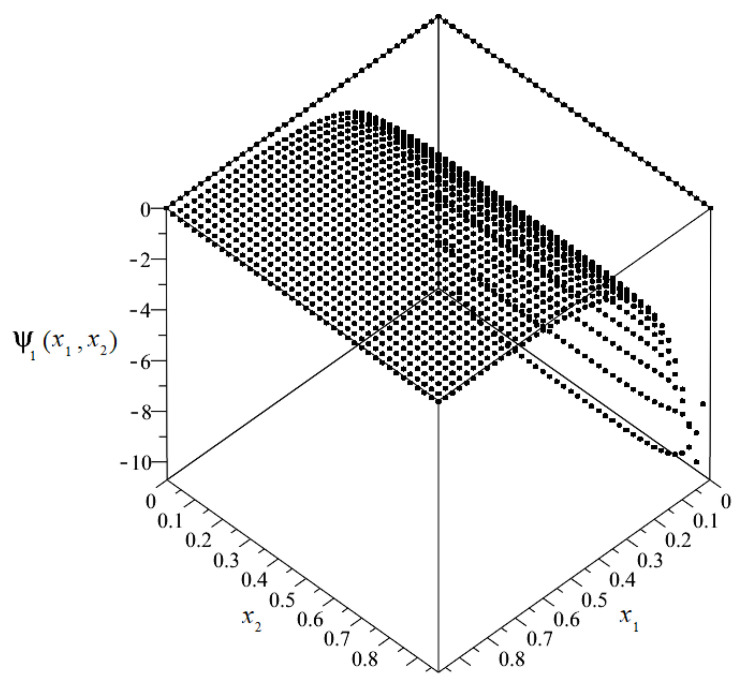
Plot of the distribution of discretized fluctuation amplitudes of temperature ψ_1_.

**Figure 11:**
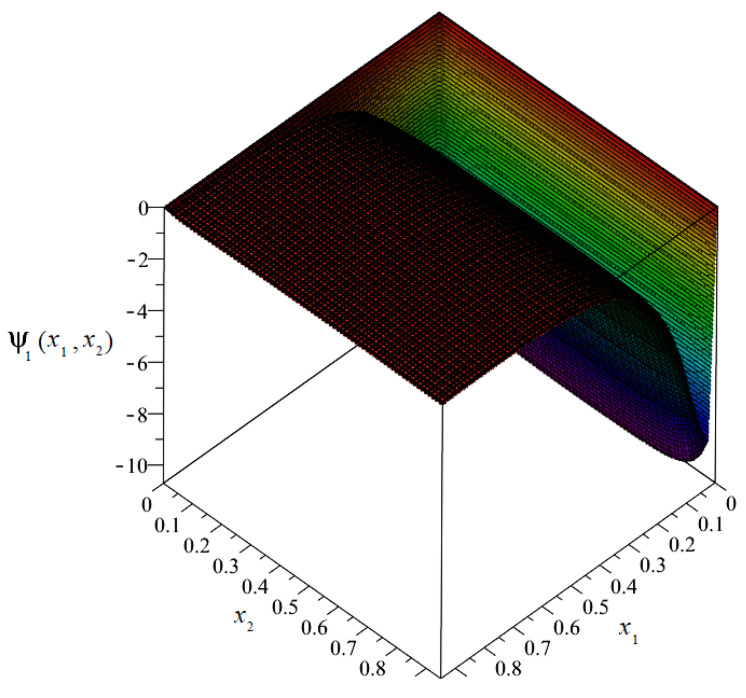
Map of the distribution of fluctuation amplitudes of temperature ψ_1_.

The plot of the distribution of discretized fluctuation amplitudes of the temperature ψ_1_ = ψ_1_(*x*_1_, *x*_2_) is shown in Figure 10, and the map in Figure 11.

**Figure 12:**
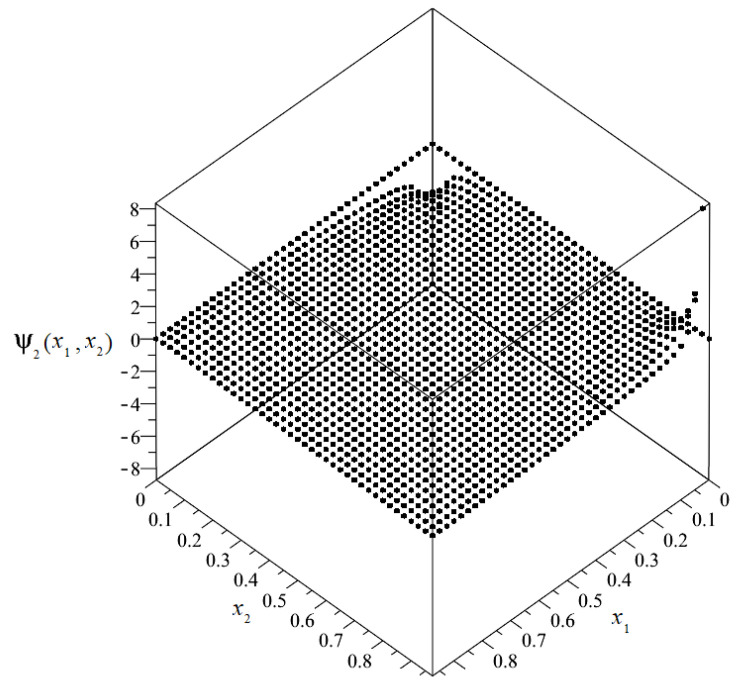
Plot of the distribution of discretized fluctuation amplitudes of temperature ψ_2_.

**Figure 13:**
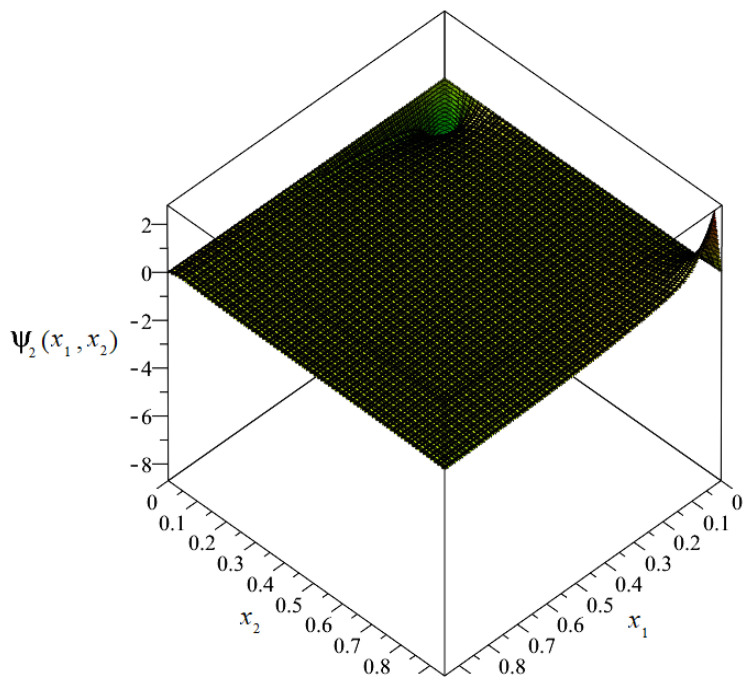
Map of the distribution of fluctuation amplitudes of temperature ψ_2_.

The plot of the distribution of discretized fluctuation amplitudes of temperature ψ_2_ = ψ_2_(*x*_1_, *x*_2_) is shown in Figure 12, and the map in Figure 13.

From the above figures, it can be observed that the values of the fluctuation amplitudes effected the values of total temperature most significantly near the surface for *x*_1_ = 0. Close by this surface, the fluctuation amplitudes represented almost 10% of the values of the macrotemperature. In the remainder of the composite, the values of the fluctuation amplitudes were close to zero and were directly related to the analyzed boundary conditions.

## 5. Conclusions

Considerations that were explored and the creation of the finite difference method algorithm allowed us to formulate the following conclusions:

1. The heat conduction equation is an equation with noncontinuous coefficients with reference to the analyzed biperiodic structure.
2. Tolerance modelling makes it possible to average the equations and consider the impacts of the microstructure size on the issues analyzed.
3. The resulting equations are equations of many variables, and it was necessary to solve them numerically.
4. The Crank–Nicolson method was used to solve the obtained system of non-uniform equations, which ensured convergence of the solutions.
5. The created algorithm is universal and may allow one to analyze a biperiodic structure composing two materials with arbitrary material properties arranged as in Figure 1.
6. The created algorithm may allow one to analyze an arbitrary value of the external temperature and the temperature of one on the surfaces of the composite.
7. Changing the boundary conditions involves modifying the algorithm, because in the finite difference method, equations are written only for nodes, where the values of the sought unknowns are not known.

## Data Availability

Not applicable.

## References

[1] Bensoussan A., Lions J.L., Papanicolay G. (1978). Asymptotic Analysis for Periodic Structures.

[2] Caillerie D., Nedelec J.C. (1984). Thin elastic and periodic plates. Math. Methods Appl. Sci..

[3] Matysiak S.J., Nagórko W. (1989). Microlocal parameters in the modelling of microperiodic plates. Ing. Arch..

[4] Matysiak S.J., Perkowski D.M. (2015). On heat conduction in periodically stratified composites with slant layering to boundaries. Therm. Sci..

[5] Santos H., Mota Soares C.M., Mota Soares C.A., Reddy J.N. (2008). A semi-analytical finite element model for the analysis of cylindrical shells made of functionally graded materials under thermal shock. Compos. Struct..

[6] Sadowski T., Ataya S., Nakonieczny K. (2009). Thermal analysis of layered FGM cylindrical plates subjected to sudden cooling process at one side. Comparison of two applied methods for problem solution. Comput. Mater. Sci..

[7] Goldberg R.K., Hopkins D.A. (1995). Thermal analysis of a functionally graded material subject to a thermal gradient using the boundary element method. Compos. Eng..

[8] Sladek J., Sladek V., Zhang C.H. (2003). Transient heat conduction analysis in functionally graded materials by the meshless local boundary integral equation method. Comput. Mater. Sci..

[9] Aboudi J., Pindera M.J., Arnold S.M. (1995). A coupled higher-order theory for functionally graded composites with martial homogenization. Compos. Eng..

[10] Aboudi J., Pindera M.J., Arnold S.M. (1999). Higher-order theory for functionally graded materials. Compos. Part B Eng..

[11] Ootao Y., Tanigawa Y. (2006). Transient thermoelastic analysis for a functionally graded hollow cylinder. J. Therm. Stresses..

[12] Woźniak C., Michalak B., Jędrysiak J. (2008). Thermomechanics of Heterogeneous Solids and Structures. Tolerance Averaging Approach.

[13] Jędrysiak J. (2010). Termomechanika Laminatów, Płyt I Powłok O Funkcyjnej Gradacji Własności.

[14] Woźniak C., Wierzbicki E. (2000). Averaging Techniques in Thermomechanics of Composite Solids. Tolerance Averaging Versus Homogenization.

[15] Carslaw H.S., Jaeger J.C. (1959). Conduction of Heat Solids.

[16] Ostrowski P., Jędrysiak J. (2021). Dependence of temperature fluctuations on randomized material properties in two-component periodic laminate. Compos. Struct..

[17] Domagalski Ł. (2018). Free and forced large amplitude vibrations of periodically inhomogeneous slender beams. Arch. Civ. Mech. Eng..

[18] Marczak J. (2021). The tolerance modelling of vibrations of periodic sandwich structures—Comparison of simple modelling approaches. Eng. Struct..

[19] Marczak J., Michalak B., Wirowski A. (2021). A multi-scale analysis of stress distribution in thin composite plates with dense system of ribs in two directions. Adv. Eng. Softw..

[20] Tomczyk B., Gołąbczak M. (2020). Tolerance and asymptotic modelling of dynamic thermoelasticity problems for thin micro-periodic cylindrical shells. Meccanica.

[21] Tomczyk B., Bagdasaryan V., Gołąbczak M., Litawska A. (2020). Stability of thin micro-periodic cylindrical shells; extended tolerance modelling. Compos. Struct..

[22] Tomczyk B., Bagdasaryan V., Gołąbczak M., Litawska A. (2021). On the modelling of stability problems for thin cylindrical shells with two-directional micro-periodic structure. Compos. Struct..

[23] Jędrysiak J. (2020). Tolerance modelling of vibrations and stability for periodic slender visco-elastic beams on a foundation with damping. Revisiting. Materials.

[24] Pazera E., Ostrowski P., Jędrysiak J. (2018). On thermoelasticity in FGL—Tolerance averaging technique. Mech. Mech. Eng..

[25] Jędrysiak J., Kaźmierczak-Sobińska M. (2020). Theoretical analysis of buckling for functionally graded thin plates with microstructure resting on an elastic foundation. Materials.

[26] Pazera E., Jędrysiak J. (2018). Thermomechanical analysis of functionally graded laminates using tolerance approach. AIP Conf. Proc..

[27] Kubacka E., Ostrowski P. (2021). Heat conduction issue in biperiodic composite using Finite Difference Method. Compos. Struct..

[28] Pazera E. (2021). Heat transfer in periodically laminated structures-third type boundary conditions. Int. J. Comput. Methods.

[29] Pletcher R.H., Tannehill J.C., Anderson D.A. (1997). Computational Fluid Mechanics and Heat Transfer.

